# Host and gut microbiome modulate the antiparasitic activity of nectar metabolites in a bumblebee pollinator

**DOI:** 10.1098/rstb.2021.0162

**Published:** 2022-06-20

**Authors:** Hauke Koch, Vita Welcome, Amy Kendal-Smith, Lucy Thursfield, Iain W. Farrell, Moses K. Langat, Mark J. F. Brown, Philip C. Stevenson

**Affiliations:** ^1^ Royal Botanic Gardens Kew, Kew Green, Richmond, Surrey TW9 3AE, UK; ^2^ Imperial College, South Kensington, London SW7 2BX, UK; ^3^ Department of Plant Sciences, University of Cambridge, Cambridge CB2 3EA, UK; ^4^ John Innes Centre, Norwich, Norfolk NR4 7UH, UK; ^5^ Centre for Ecology, Evolution and Behaviour, Department of Biological Sciences, Royal Holloway University of London, Egham, Surrey TW20 0EX, UK; ^6^ Natural Resources Institute, University of Greenwich, Greenwich, Kent ME4 4TB, UK

**Keywords:** bee health, disease, phytochemistry, host–parasite ecology, gut microbiota, Trypanosomatidae

## Abstract

Antimicrobial nectar secondary metabolites can support pollinator health by preventing or reducing parasite infections. To better understand the outcome of nectar metabolite–parasite interactions in pollinators, we determined whether the antiparasitic activity was altered through chemical modification by the host or resident microbiome during gut passage. We investigated this interaction with linden (*Tilia* spp.) and strawberry tree (*Arbutus unedo*) nectar compounds*.* Unedone from *A. unedo* nectar inhibited the common bumblebee gut parasite *Crithidia bombi in vitro* and in *Bombus terrestris* gynes. A compound in *Tilia* nectar, 1-[4-(1-hydroxy-1-methylethyl)-1,3-cyclohexadiene-1-carboxylate]-6-*O*-β-d-glucopyranosyl-β-d-glucopyranose (tiliaside), showed no inhibition *in vitro* at naturally occurring concentrations but reduced *C. bombi* infections of *B. terrestris* workers. Independent of microbiome status, tiliaside was deglycosylated during gut passage, thereby increasing its antiparasitic activity in the hindgut, the site of *C. bombi* infections*.* Conversely, unedone was first glycosylated in the midgut without influence of the microbiome to unedone-8-*O*-β-d-glucoside, rendering it inactive against *C. bombi*, but subsequently deglycosylated by the microbiome in the hindgut, restoring its activity. We therefore show that conversion of nectar metabolites by either the host or the microbiome modulates antiparasitic activity of nectar metabolites.

This article is part of the theme issue ‘Natural processes influencing pollinator health: from chemistry to landscapes’.

## 1. Introduction

The health of wild pollinators is under threat from parasites through a variety of anthropogenic factors, including the potential introduction of parasites into new geographical areas by global trade [1,2], spill-over of emerging infectious diseases from managed pollinators like honeybees [3,4] or through additive or synergistic effects between parasites and other man-made stressors like pesticides [5,6]. Dietary secondary plant compounds naturally occurring in nectar or pollen could ameliorate these threats to pollinator health via increased tolerance, prevention or reduction of infections [7–11]. Understanding the role of different foraging plants for pollinator diseases may thus present a promising avenue to promote pollinator health, for example by protecting natural habitats with key plant species [9] or promoting forage plants with health benefits through seed mixes in agricultural environments [11]. However, we still lack a detailed understanding of the factors that determine the effects of dietary phytochemicals on parasites of pollinators within the host. Our ability to predict the outcomes of the diversity of possible phytochemical–pollinator–parasite interactions in the wild is therefore limited [12]. Indeed, the effect of secondary nectar metabolites on parasites in pollinator hosts has, in some cases, been inconsistent or contradictory between studies in the same host–parasite system (e.g. [7,13]) and may be affected by host genotypes or environmental conditions like temperature and food composition [13,14]. *In vitro* screens of nectar and pollen phytochemicals can provide insights into direct effects on pollinator parasites in culture, for example showing synergistic effects between compounds [15], variation in resistance against compounds between different parasite genotypes [16] or effects on parasite cell morphology [9]. However, without studying the fate of dietary phytochemicals in the host, we cannot establish whether these simplified *in vitro* experiments reflect conditions experienced by parasites *in vivo* and thus whether they are ultimately relevant in ecological contexts [9].

The fate of phytochemicals after ingestion by pollinators before reaching parasite infection sites likely influences their antiparasitic effect. Plant compounds may be chemically transformed during passage through the bee gut [17]. This could either increase or decrease their activity against parasites. Koch *et al*. [9], for example, found that callunene from heather nectar can reduce the likelihood of infections with the common trypanosomatid gut parasite *Crithidia bombi* in bumblebees (*Bombus terrestris*) when parasite cells were exposed for a short time in the crop. However, callunene concentration sharply declined during gut passage and did not reach the site of infection in the hindgut. Consequently, existing infections remained unaffected by callunene ingestion. Although dietary phyotchemicals may not reach internal parasite infections in their ingested form, modification of these compounds post-ingestion could modulate their subsequent impact on parasites. However, we currently lack an understanding of the processes underlying these changes to phytochemical structures and concentrations.

Chemical modification of dietary secondary metabolites in the bee gut can be caused by host enzymes secreted into the gut [18]. Bees produce a range of enzymes that can metabolize dietary secondary metabolites, including cytochrome P450 monooxygenases (P450s) and glutathione transferases [18,19]. However, detoxification gene diversity is reduced in honeybees and bumblebees compared to other insects [18,20]. The resident gut microbiome of social bees (see [21]) may therefore play an important additional role in metabolizing dietary secondary compounds. Kešnerová *et al*. [22], for example, showed that the microbiome metabolized flavonoid glycosides in the honeybee gut, but the extent and functional relevance of metabolic transformation of secondary metabolites by the bee gut microbiome is not well understood. The presence and composition of the bacterial microbiome in bumblebees has previously been shown to influence parasite infections with the gut parasite *C. bombi* [23–25]. The mechanisms for this health benefit remain unclear but could include changes to the chemical environment in the bee gut.

Here, we studied this interaction between the pollinator host, nectar phytochemicals, parasites and the microbiome. Following the discovery of *in vitro* activity of monofloral honey extracts from linden (*Tilia* spp.) and strawberry tree (*Arbutus unedo*) against *C. bombi* described in Koch *et al*. [9], we investigated three key issues. First, we tested the antiparasitic activity of secondary metabolites from the nectar of these tree species through *in vitro* and *in vivo* experiments with the important European bumblebee pollinator species *B. terrestris*. Second, we investigated if chemical transformation of nectar secondary metabolites post-ingestion modulated their antiparasitic activity. Third, we tested the role of the host and the gut microbiome in the transformation of ingested nectar secondary metabolites.

## 2. Methods

### (a) Nectar analysis

Nectar samples were collected from *A. unedo* L. and *Tilia tomentosa* Moench trees growing at the Royal Botanic Gardens, Kew (Richmond, Surrey, UK; RBG Kew) in October 2019 (*A. unedo*) and July 2015 (*T. tomentosa*). Flowers were gauze-bagged to prevent removal of nectar by bees, and nectar was collected after 1 day with 10 µl glass capillaries (Drummond Scientific, Broomall, USA). *Bombus terrestris* gynes (i.e. potential queens) foraging on *A. unedo* at RBG Kew were caught in October 2019, and nectar-filled crops dissected out for chemical analysis of contents. All samples were weighed (Mettler Toledo Balance XS105), extracted in 80% methanol (including macerating bumblebee crop samples with plastic pestles), briefly vortexed, held in the dark for 24 h at room temperature, briefly vortexed again, and centrifuged for 2 min at 3000 rpm (= 845*g*), and supernatants stored at −20°C until further analysis (see also methods in [9]).

Extracts were analysed via HPLC-MS (Velos-Pro; Thermo Fisher Scientific; with a photodiode array (PDA)) and high-resolution electrospray-ionization mass spectrometry (HR-ESI-MS) on a Thermo Fisher Scientific LTQ Orbitrap, with 5 µl injection volume onto a Phenomenex Luna C18(2) column (150 × 3 mm, 3 µm particle size) held at 30°C, and a linear mobile phase gradient of 10–100% aqueous MeOH containing 0.1% formic acid over 20 min. We focused our analyses on a major secondary metabolite from *Tilia* honey: 1-[4-(1-hydroxy-1-methylethyl)-1,3-cyclohexadiene-1-carboxylate]-6-*O*-β-d-glucopyranosyl-β-d-glucopyranose [26,27] (to which we assign the trivial name tiliaside) and a major component of *A. unedo* honey: the isoprenoid unedone (2-(1,2-dihydroxypropyl)-4,4,8-trimethyl-1-oxaspiro[2.5]oct-7-en-6-one) [28]. We quantified unedone and tiliaside with peak areas recorded at their UV absorbance maxima (unedone: 245 nm; tiliaside: 308 nm) and compared with calibration curves from pure standards between 1 and 1000 ppm (see electronic supplementary material, data).

### (b) Compound isolation and identification

Nectar compounds were isolated from monofloral honey of *A. unedo* (source: Wild about Honey, Portugal) or *Tilia* sp. (source: Tesco, UK; honey origin: Romania) respectively, as monofloral honey offers a source for bulk quantities of nectar compounds that are often similar to the chemical composition of the nectar from which it is derived [9].

To isolate unedone, *A. unedo* honey was dissolved in ultra-pure water (Milli-Q; Sigma, St Louis, MO) in a 1 : 2 (weight/weight) ratio. The diluted honey was then mixed with ethyl acetate in a 1 : 1 ratio (volume/volume). The mixture was shaken in a separating funnel until an emulsion was formed and left to separate overnight. The ethyl acetate layer was removed and dried on a rotary evaporator. Extracts were re-dissolved in 80% methanol and partitioned on a flash chromatography system (Biotage Isolera One; Biotage, Sweden) using a SNAP Ultra C18 cartridge (water–methanol gradient: 5% methanol for 1.5 column volumes (CVs); 14% methanol: 1.5 CVs; 31% methanol: 5.5 CVs; 100% methanol: 1 CV). Unedone eluted at 5 CVs, and collection was guided by monitoring UV absorbance at 245 nm. Solvent was removed on a rotary evaporator, purity evaluated by ^1^H NMR comparing with chemical shifts in Tuberoso *et al*. [28], and extract stored at −20°C until further use.

A previously undescribed glycosylated derivate of unedone, unedone-8-*O*-β-d-glucoside, was isolated from *B. terrestris* gynes’ faeces. For this, gynes collected at RBG Kew were housed in individual plastic boxes and fed with 50% apiinvert sugar syrup containing 3.79 mM (910 ppm) unedone. Faecal material was harvested daily, either by collecting faeces with a 10 µl glass capillary from gynes periodically placed in plastic tubes or by rinsing filter paper placed into the bottom of cages with ethanol. Faecal material was combined, filtered and dried down, and unedone-8-*O*-β-d-glucoside was purified. First, faecal material was dissolved in H_2_O, combined with an equal volume of ethyl acetate, and shaken up in a separation funnel to an emulsion, and the ethyl acetate layer was collected after the emulsion had separated. The ethyl acetate was dried down, and resuspended in methanol, and unedone-8-*O*-β-d-glucoside was isolated on a flash chromatography system (Biotage Isolera One; Biotage, Sweden) using a SNAP Ultra C18 cartridge (water–methanol gradient: 5–30% methanol linear gradient: 7 CVs; 30% methanol: 4 CVs). Unedone-8-*O*-β-d-glucoside eluted at 8.9 CVs. To elucidate the structure, unedone-8-*O*-β-d-glucoside was dissolved in CDCl_3_ and analysed by nuclear magnetic resonance (NMR) spectroscopy (400 MHz Bruker Avance; Bruker, Billerica, MA) using 1D (^1^H, ^13^C and DEPT (distortionless enhancement by polarization transfer)) and 2D (^1^H-^1^H COSY (correlation spectroscopy), ^1^H-^1^H ROESY (rotating-frame nuclear Overhauser effect spectroscopy), ^1^H-^13^C HSQC (heteronuclear single quantum coherence spectroscopy) and ^1^H-^13^C HMBC (heteronuclear multiple bond correlation)) NMR spectroscopic analysis.

For extraction of tiliaside, *Tilia* honey was extracted in ethanol by mixing one part honey with two parts ethanol (by weight) in a conical flask, stirring the mix with a glass rod for 5 min and placing the mix on an orbital shaker for 1 h, with additional stirring every 10 min. The ethanol was collected (leaving most of the sugar behind) and dried on a rotary evaporator until all the solvent had been removed. The extract was partitioned via flash chromatography (see above, but with the following water–methanol gradient: 10–15% methanol: 5 CVs; 30% methanol: 3 CVs; 45% methanol: 2.5 CVs; 75–100% methanol: 1.5 CVs). Tiliaside eluted at around 6 CVs, and the corresponding aglycone at around 8.5 CVs; collection was guided by monitoring UV absorbance at 308 nm. Tiliaside and the corresponding aglycone were further purified by semi-preparative HPLC on a Waters (UK) LC system (600E pump, 996 PDA detector; Phenomenex Luna C18 column: 150 × 10 mm, 10 µm particle size), and purity verified by ^1^H NMR, comparing with chemical shifts in Frérot *et al*. [27]. The structures for tiliaside and the aglycone of tiliaside were determined *ab initio* using NMR and MS data, and by comparison with the reported data from Frérot *et al*. [27] (see electronic supplementary material).

### (c) *In vivo* experiments

For unedone *in vivo* experiments, *B. terrestris* gynes were collected in autumn 2018 at RBG Kew. We selected gynes for the experiment, as these are the dominant caste foraging on *A. unedo* at the time of flowering (H. Koch 2016–2021, personal observation; [29]). Individual gynes were housed in plastic boxes and fed with 50% apiinvert sugar syrup (Apiinvert, Südzucker, Germany) and honeybee-collected polyfloral pollen (Biobest, Belgium). Faeces of gynes were screened microscopically for parasite infections (*Crithidia*, *Nosema*, *Apicystis*) on the day of capture and after two weeks in the laboratory, and infected gynes were excluded. Uninfected gynes were randomly assigned to two treatments: a control treatment receiving 50% apiinvert sugar syrup, and a unedone treatment receiving 50% apiinvert sugar syrup containing 3.79 mM (910 ppm) unedone (around the limit of solubility of unedone in the diet, and below the average concentration measured in *B. terrestris* gyne crops foraging on *Arbutus* at RBG Kew). Both groups also received polyfloral pollen *ad libitum* (Biobest, Belgium). For *C. bombi* inoculations, gynes were placed in individual plastic vials and deprived of food, then after 6 h fed 15 µl of an inoculum containing 15 000 cells of *C. bombi* from a laboratory *in vitro* culture (for source see strain details in Koch *et al*. [9]) mixed 1 : 2 with 50% apiinvert sugar syrup. After feeding on the inoculum, gynes were put back into their cages and received either the control or unedone diet for 7 days. As prevention of infection in gynes before hibernation can be expected to have major fitness benefits [30], we designed this experiment to test if feeding on unedone can reduce the risk of infections to gynes. After 7 days, faeces were sampled from gynes, and *C. bombi* cell concentrations were determined with a Neubauer improved haemocytometer and phase contrast microscope (Zeiss Photomicroscope III; Carl Zeiss AG, Germany) at 640× magnification. We used an analysis of variance (ANOVA) with log-transformed *C. bombi* faecal concentrations as dependent variable and diet treatment groups as independent variable in R [19]. To test for effects of diet treatment on infection success, we used a *χ*^2^-test in R [31], scoring gynes without *C. bombi* in the faeces as uninfected (0) and gynes with any concentration of *C. bombi* as infected (1).

For the tiliaside *in vivo* experiment, *B. terrestris* workers were removed from colonies originating from wild-caught queens at RBG Kew, housed in individual plastic boxes with 50% apiinvert sugar syrup and *ad libitum* polyfloral pollen (Biobest, Belgium), and infected with a laboratory strain of *C. bombi* (see above, inoculum of 15 000 cells)*.* After 7 days, infections were verified by microscopic examination of faeces, and uninfected individuals excluded from the experiment. Workers were then randomly assigned to feed on either a 50% apiinvert sugar syrup control or 9.88 mM (5000 ppm) of tiliaside in 50% apiinvert sugar syrup. As many workers can be expected to be infected with *C. bombi* during the flowering period of *Tilia* in summer, but will have energetic costs from infections [32], we tested in this experiment if the feeding on tiliaside can reduce existing parasite loads. After 7 days, *C. bombi* infection levels were quantified microscopically from faecal samples, and log-transformed faecal *C. bombi* concentrations analysed via a linear mixed-effects model (lme) with treatment as fixed effect and colony as random effect with the function lme of the package nlme in R [33]. Gut fragments (crop, midgut, hindgut) were dissected from the bumblebees feeding on tiliaside in this experiment and analysed together with faecal samples for the degree of conversion of tiliaside to the corresponding aglycone of tiliaside (for analytical procedure, see §4e). We tested for significant differences between proportions of the aglycone of tiliaside to tiliaside in the different gut segments with an ANOVA with logit-transformed proportions and Tukey's HSD test for pairwise comparisons in R [31].

### (d) *In vitro* experiments

*In vitro* testing of all compounds was conducted following methods in Koch *et al*. [9]. Briefly, a *C. bombi* strain isolated from *B. terrestris* (for details see [9]) was grown in standard *Crithidia* liquid medium [34] at 28°C and 3% CO_2_. Inhibition of *C. bombi* growth was tested in 96-well tissue culture plates (Eppendorf, Germany) with compounds dissolved in the culture medium in a dilution series with twofold concentration changes per step (6.25 to 0.78 mmol l^−1^ for unedone/unedone-8-*O*-β-d-glucoside; 20 to 2.5 mmol l^−1^ for tiliaside and the corresponding aglycone). To facilitate solubilizing compounds, we first dissolved compounds in methanol and added dissolved compounds to the culture medium to make up a final concentration of 1% methanol. Choice of concentration ranges reflected concentrations found in nectar but had to be limited to a maximum of 6.25 mmol l^−1^ for unedone (1500 ppm), below the maximum natural concentration in nectar found in our study, but at the limit of solubility in the culture medium. The test medium with 1% methanol was included as negative control. An aliquot of 20 µl of a 1000 cells µl^−1^ *C. bombi* culture was mixed with 180 µl of test medium in each cell. After incubation at 28°C and 3% CO_2_ for 7 days, *C. bombi* cell concentrations were determined microscopically with a Neubauer improved haemocytometer and phase contrast microscope (Zeiss Photomicroscope III; Carl Zeiss AG, Germany) at 640x magnification. Dose–response curves and estimates of the 50% effective dose (ED50) were calculated with the drm function of the package drc in R [35], using a three-parameter log-logistic model (fct = LL.3).

### (e) Microbiome experiment

Previous experiments by Koch & Schmid-Hempel [23] suggest that newly emerged bumblebees lack the core resident microbiome and acquire it through social contact in the colony post-emergence. To create microbiome-depleted or -colonized bumblebees, we here followed procedures outlined in Koch & Schmid-Hempel [23] but expanded on their procedures by surface-sterilizing pupae and maintaining newly emerged bumblebees in sterile, air-filtered environments to prevent subsequent environmental contamination: *B. terrestris* worker and male pupae were carefully removed from cocoons of laboratory colonies (Biobest). Cocoons were opened and pupae removed with superfine stainless-steel forceps (flame-sterilized between individuals) from cocoons at a stage of development with the cuticula mostly or completely dark (corresponding to pupal stages P14–P16 in [36]). Pupae were then transferred to a laminar flow hood, immersed in sterile phosphate-buffered saline (PBS) with bleach (0.2% calcium hypochlorite, freshly prepared on the day) for 1 min for surface sterilization, rinsed in autoclaved PBS twice to wash off bleach, and placed onto autoclaved filter paper to remove the PBS. Bumblebee pupae were then incubated in sterile polypropylene containers with a cover containing a filter strip allowing for sterile gas exchange (OS140BOX, round model, 140 mm height, 90 mm diameter; Duchefa Biochemie, Haarlem, NL) at 30°C and 80% humidity. During all stages of the experiment, containers were only opened under a laminar flow hood to prevent microbial contamination, and all handling of bumblebees or contents of the container was conducted with sterilized implements. Once every morning, containers were checked for emergence of individuals, and bumblebees were removed, kept for 3 h in sterile 90 mm Petri dishes and fed either 15 µl sterile 50% apiinvert sugar syrup (control) or 15 µl of a 2 : 1 mix of 50% apiinvert sugar syrup mixed with freshly collected faeces from five workers within the mother colony (to transplant the gut microbiome, see [23]). Individuals that fed on the inoculum within 1 h were then placed back into their container and kept at 26°C. Sterile pollen was provided to all individuals from polyfloral honeybee-collected pollen (Biobest, Belgium) that was ground to a powder and soaked in 70% ethanol for 1 h. Pollen was then spread out in a thin layer in sterile glass dishes in a laminar flow hood, and air-dried for 24 h to remove the solvent. Aliquots of approx 0.5 g sterilized pollen were placed into small containers made from 1.5 ml Eppendorf tubes cut in half and using the inverted top half with the lid closed. Pollen diet aliquots were stored at −20°C until use. All individuals were first fed on filter-sterilized 50% apiinvert sugar syrup and pollen for 7 days to allow establishment of the microbiome, and then split to receive either 3.79 mmol l^−1^ (910 ppm) unedone or 9.88 mmol l^−1^ (5000 ppm) tiliaside in 50% sugar syrup for a further 2 days. Diets were sterile-filtered (Stericup sterile vacuum filtration system; Millipore, Burlington, USA) and 5 ml of sterile diet was presented to each bumblebee in 7 ml inverted Sterilin polystyrene containers (Sterilin Ltd, UK) with small holes over the rim of the lid for access. After 2 days on the unedone or tiliaside diet, bumblebees were removed from containers, chilled on ice, decapitated, and dissected under a laminar flow hood with sterilized implements, and gut fragments (crop, midgut, hindgut) were placed individually into sterile, weighed 1.5 ml Eppendorf tubes. Gut weights were determined on a Mettler Toledo Balance XS105 scale.

Gut fragments were macerated with sterile plastic pestles in 30 µl sterile 1/8 strength Ringer's solution and 5 µl was transferred into a separate 1.5 ml Eppendorf tube for culturing. The 5 µl gut macerates were serially diluted by a factor of 10 for three times in sterile 1/8 strength Ringer's solution, and 5 µl of each dilution step was plated out on brain–heart infusion agar plates. Plates were incubated for 5 days at 35°C and 5% CO_2_ and colony-forming units (CFU) counted. We note that some bacterial members of the bumblebee microbiome are fastidious and would show poor or no growth under our culturing conditions, and consequently the absence of microbial growth on the culture plates does not necessarily indicate microbial sterility. A 50 µl ethanol aliquot was mixed with the remaining original 25 µl macerated gut for metabolite extraction, sonicated for 5 min and left for 24 h at room temperature. Suspensions were then spun down (3000 rpm (= 845*g*), 3 min) and supernatants collected into autosampler vials for HPLC-MS analysis. Samples were analysed by HR-ESI-MS on a Thermo Fisher Scientific LTQ Orbitrap with PDA detector. Target compound peaks were verified by mass of the pseudomolecular ions in positive mode and by comparison with standards of pure compounds. UV absorbance of the glycosides and corresponding aglycones (308 nm for tiliaside and aglycone of tiliaside; 245 nm for unedone and unedone-8-*O*-β-d-glucoside) was measured to estimate ratios of glycosides to aglycones in the different gut segments.

## 3. Results

### (a) Presence of unedone and tiliaside in nectar

Unedone, previously characterized from *Arbutus* honey [28], was found in the HPLC-MS analysis of strawberry tree (*A. unedo*) nectar, verified with a unedone standard isolated from *Arbutus* honey (see Methods; for NMR data see electronic supplementary material, figure S8), showing a matching *m*/*z* 241 pseudomolecular ion [M + H]^+^ in positive mode at retention time 9.35 min with a UV absorbance maximum of 245 nm. The accurate mass of the *m*/*z* 241 pseudomolecular ion [M + H]^+^ in positive mode analysed via HR-ESI-MS furthermore matched the predicted mass from the molecular formula of the [M + H]^+^ ion (observed *m*/*z* 241.1435; Δppm 0.350 versus expected for C_13_H_21_O_4_) and had a matching MS2 spectrum (see electronic supplementary material, figure S10). Quantification using peak areas of UV absorbance gave a nectar concentration at an average of 14.66 mmol l^−1^ (3518 ppm) unedone (*n* = 7; range: 6.34−35.8 mmol l^−1^). Crop contents of *B. terrestris* gynes foraging on *A. unedo* had an average of 9.15 mmol l^−1^ (2195 ppm) unedone (*n* = 3; range 7.48−11.42 mmol l^−1^).

*Tilia tomentosa* nectar contained tiliaside. Tiliaside was verified by comparison with a pure standard isolated from *Tilia* honey (see §2; for NMR data see electronic supplementary material, table S2 and figure S11; see also [27]) and accurate mass of the main pseudomolecular ions in positive mode analysed via HR-ESI-MS ([M + NH_4_]^+^ (*m*/*z* = 524.2336; Δppm −0.356 versus expected for C_22_H_38_O_13_N) and [M + H]^+^ (*m*/*z* = 507.2072; Δppm 0.044 versus expected for C_22_H_35_O_13_)), as well as the matching MS2 spectrum (electronic supplementary material, figure S13). Quantification using UV absorbance (308 nm) peak area gave an average concentration of 16.74 mmol l^−1^ (8469 ppm; *n* = 3). This is similar to the 11.86 mmol l^−1^ (6000 ppm = 0.6%) of tiliaside reported by Frérot *et al*. [27] in Swiss linden honey (likely from *Tilia cordata* or *Tilia platyphyllos*).

### (b) *In vivo* effects of unedone and tiliaside and conversion during gut passage

**Figure 1.**
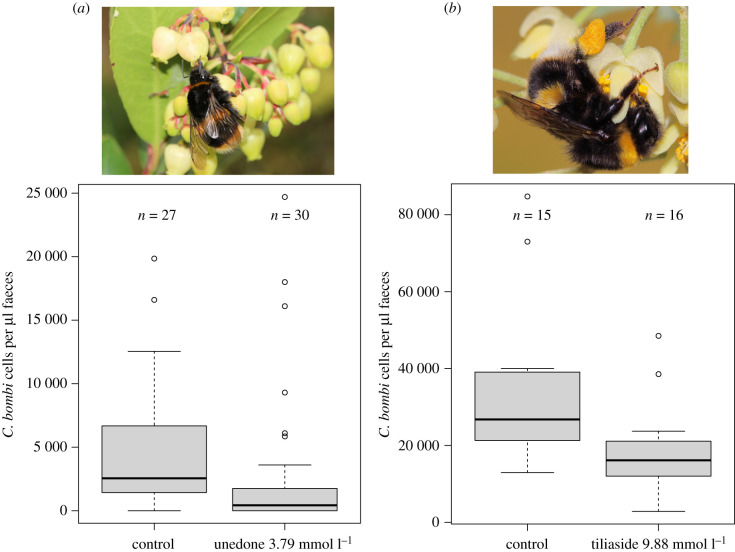
Activity of dietary unedone and tiliaside against *Crithidia bombi* in *Bombus terrestris*. (*a*) Boxplot of faecal concentration of *C. bombi* in *B. terrestris* gynes 7 days post-infection when feeding on control or unedone (3.79 mmol l^−1^) diets. Image: *B. terrestris* gyne foraging on *A. unedo* at Kew Gardens, UK. (*b*) Boxplot of faecal concentration of *C. bombi* in *B. terrestris* workers with pre-established infections after feeding for 7 days on control or tiliaside (9.88 mmol l^−1^) diets. Image: *B. terrestris* worker foraging on *Tilia tomentosa* flower at Kew Gardens, UK. (Online version in colour.)

*Bombus terrestris* gynes feeding on 3.79 mmol l^−1^ (910 ppm) unedone from *A. unedo* had lower infection levels in faeces samples 7 days after inoculation compared with the sugar water-fed control group (figure 1*a*; ANOVA: *F*_1,55_ = 15.02, *p* = 0.00029) and were less likely to have developed an infection (*χ*^2^ = 10.63, *p* = 0.0011; 96% infected in control group versus 60% infected in unedone group; *n* = 57). Tiliaside from *Tilia* nectar reduced *C. bombi* infection levels in faeces of *B. terrestris* workers with pre-established *C. bombi* infections after feeding on 9.88 mmol l^−1^ (5000 ppm) tiliaside for 7 days, compared with the sugar water-fed control group (figure 1*b*; lme: *F*_1,26_ = 9.8, *p* = 0.0042).

HR-ESI-MS analyses showed that tiliaside was partially deglycosylated during gut passage in *B. terrestris* workers. The proportion of the corresponding aglycone, 4-(1-hydroxy-1-methylethyl)-1,3-cyclohexadiene-1-carboxylic acid (observed [M + H]^+^ *m*/*z* = 183.1016, Δppm 0.050 versus expected for C_10_H_15_O_3_; for MS2 spectrum see electronic supplementary material, figure S14; for NMR data see electronic supplementary material, table S2 and figure S12), to tiliaside was low in the crop (average 9%; *n* = 15) and midgut (5%; *n* = 15), but significantly increased in the hindgut (18%; *n* = 15) and faeces (27%; *n* = 16) (Tukey's HSD test; see electronic supplementary material, figure S1).

**Figure 2.**
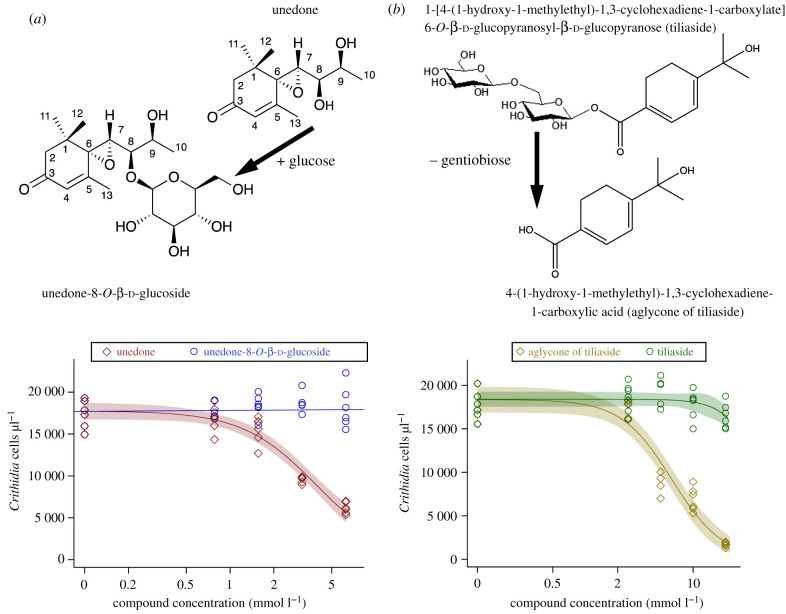
*In vitro* activity test of *Tilia* and *Arbutus* compounds against *C. bombi*, and chemical structures of compounds investigated in this study. (*a*) Top: structures of unedone and unedone-8-*O*-β-d-glucoside (numbered carbons for NMR resonances in electronic supplementary material, table S1). Bottom: *in vitro* assay comparing inhibition of *C. bombi* by unedone and unedone-8-*O*-β-d-glucoside at equimolar concentrations. *y*-axis: *C. bombi* cell concentrations in culture after 7 days. Dose–response curve (three-parameter log-logistic model) fitted to unedone responses, with 95% CIs shaded around regression curve. Linear regression line fitted to unedone-8-*O*-β-d-glucoside responses shows no decline with increased dose. (*b*) Top: structures of tiliaside and the aglycone of tiliaside. Bottom: *in vitro* assay comparing inhibition of *C. bombi* by the aglycone of tiliaside and tiliaside at equimolar concentrations. *y*-axis: *C. bombi* cell concentrations in culture after 7 days. Dose–response curve (three-parameter log-logistic model) fitted to responses to both compounds, with 95% CIs shaded around regression curve. (Online version in colour.)

A peak with similar UV absorbance to unedone (peak 245 nm) but higher mass (observed [H + M]^+^ *m*/*z* = 403.1960, Δppm −0.617 versus expected for C_19_H_31_O_9_; for MS2 spectrum see electronic supplementary material, figure S9), consistent with a hexoside of unedone, was recorded in an extract of faeces from bumblebee gynes fed on diets containing unedone. NMR spectroscopy of the compound purified from faeces of *B. terrrestris* gynes fed on unedone showed it to be a previously undescribed compound, unedone-8-*O*-β-d-glucoside (figure 2*a*; electronic supplementary material, figures S2–S7 and table S1). The structure of the compound was determined as a mono-glycosylated derivative of the known unedone [28], using 1D (^1^H, ^13^C and DEPT), and 2D (^1^H-^1^H-COSY, ^1^H-^1^H ROESY, ^1^H-^13^C HSQC and ^1^H-^13^C HMBC) NMR spectroscopic analysis and MS. The ^13^C NMR data for unedone [28], shown in electronic supplementary material, table S1 (column 1a), were comparable with those of the aglycone of this compound, except for C-8, which was deshielded at *δ*_C_ 82.3 (unedone gives *δ*_C_ 72.1). The analysis of the HMBC (see electronic supplementary material, table S1) showed that the C-8 position was substituted with a glycosidic moiety, and comparison of the reported data for β-glucose [37] (electronic supplementary material, table S1), and those of this compound showed them to be similar; hence, the sugar unit was tentatively assigned as β-glucose. Use of a model and ^1^H-^1^H ROESY allowed the assignment of the relative configuration of the compound, with an epoxy group on one side and the hydroxy group and glucose groups on the other face of the molecule, as shown in electronic supplementary material, figure S3, and the compound was determined to be unedone-8-*O*-β-d-glucoside.

### (c) *In vitro* activity of unedone versus unedone-8-*O*-β-d-glucoside and tiliaside versus aglycone of tiliaside

Unedone inhibited *C. bombi* at 3.125 and 6.25 mmol l^−1^ (considerably below the average of 14.66 mmol l^−1^ measured in nectar) (figure 2*a*)*.* We estimated the ED_50_ for unedone at 3.94 mmol l^−1^. By contrast, unedone-8-*O*-β-d-glucoside showed no inhibition of *C. bombi in vitro* up to a concentration of 6.25 mmol l^−1^, the highest concentration that was feasible for us to measure. This suggests glycosylation of unedone in the bumblebee gut will remove its antiparasitic activity. Tiliaside from linden nectar had no activity against *C. bombi* up to 20 mmol l^−1^ (higher than the concentration found in *Tilia* nectar by us), while the corresponding aglycone of tiliaside reduced *C. bombi* growth at concentrations from 5 to 20 mmol l^−1^ with an estimated ED_50_ of 6.35 mmol l^−1^ (figure 2*b*). Conversion of the approximately 17 mmol l^−1^ tiliaside in nectar into the corresponding aglycone would therefore lead to strong inhibition of *C. bombi*.

### (d) Microbiome experiments

Microbiome-inoculated bumblebee workers had an average of 1.9 × 10^6^ CFU, and males 5.9 × 10^6^ CFU per hindgut on BHI agar, with all individuals showing microbial growth. Hindguts of microbiome-depleted males and workers had no microbial growth on BHI agar, even when plated out at the lowest dilution step (1/60th of total hindgut plated out), except for a single individual each of the males and workers having 1 CFU at the lowest dilution step (possible contaminant). This suggests that our experimental protocol was effective for reducing microbial colonization of the gut and restoring it in the microbiome-inoculated individuals.

**Figure 3.**
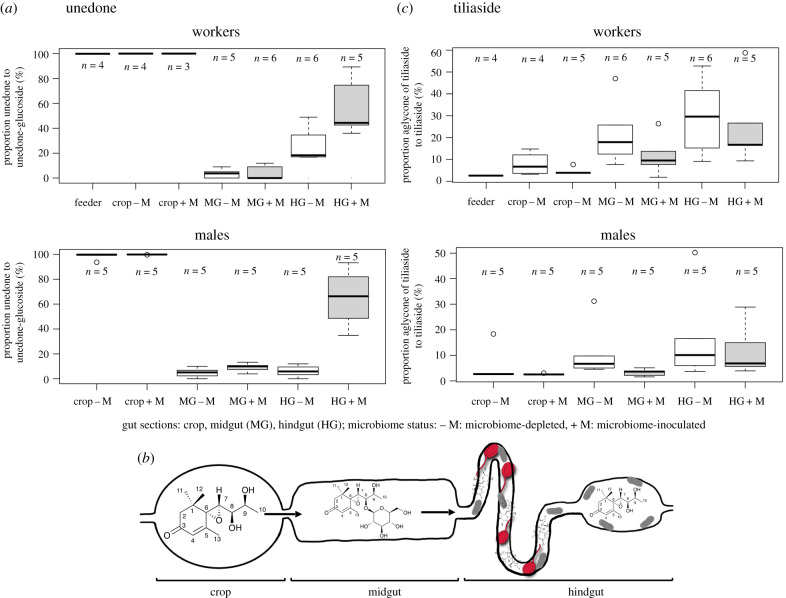
Conversion of unedone and tiliaside during gut passage in microbiome-depleted or -inoculated individuals. (*a*) Boxplot of proportion of unedone to unedone-8-*O*-β-d-glucoside during gut passage in workers (top) and males (bottom) for microbiome-depleted (−M: white boxes) or -inoculated individuals (+M: grey-shaded boxes). (*b*) Schematic of proposed transformation of unedone during gut passage: unedone is ingested into the crop, transformed by the bumblebee into unedone-8-*O*-β-d-glucoside in the midgut and deglycosylated again into unedone by gut microbiome (grey rods) in the hindgut, where it can inhibit *C. bombi* parasites (red). (*c*) Boxplot of proportion of the aglycone of tiliaside to tiliaside during gut passage in workers (top) and males (bottom) of microbiome-depleted (−M: white boxes) or -inoculated individuals (+M: grey-shaded boxes). (Online version in colour.)

Only unedone, and not its glucoside, was detected in the crop of both workers and males, independent of microbiome status (figure 3*a*). By contrast, in both workers and males, the unedone-8-*O*-β-d-glucoside was highly dominant (greater than 90%) over unedone in the midgut, suggesting that unedone is glycosylated in this gut compartment (figure 3*a*). No difference was apparent between microbiome-depleted and microbiome-inoculated individuals, suggesting that the glycosylation likely derives from enzymes of the bumblebee secreted into the midgut. A reversion to a higher proportion of unedone to unedone-8-*O*-β-d-glucoside was recorded in the hindgut of microbiome-inoculated males and workers, suggesting partial deglycosylation in this gut compartment. However, the reversion to unedone was not apparent in microbiome-depleted males and limited in microbiome-depleted workers (figure 3*a*), suggesting that the deglycosylation of unedone-8-*O*-β-d-glucoside was caused by the hindgut microbiome. A schematic of the fate of unedone in the gut is given in figure 3*b*.

The proportion of tiliaside to its aglycone increased from the crop to the midgut and hindgut (figure 3*c*). Neither workers nor males showed apparent differences in the proportion of tiliaside to its aglycone between microbiome-inoculated and microbiome-depleted individuals (figure 3*c*), implying that the microbiome did not play a major role in this conversion, but that it is likely host-induced.

## 4. Discussion

We demonstrate that the conversion of nectar secondary metabolites in the gut of bumblebees can modulate their activity against the common gut parasite *C. bombi*. Importantly, the activity of secondary compounds can be both increased and decreased during gut passage, showing that a better understanding of the fate of nectar or pollen secondary metabolites after ingestion is necessary to determine their effects on parasites of pollinators. Simplified studies of secondary metabolites from pollen or nectar in *in vitro* assays alone may therefore not accurately predict their interactions and effects on parasites in the host, as they may either over- or underestimate effects. Our findings furthermore show that the antiparasitic activity of dietary secondary metabolites in a host can be altered by chemical changes induced by either the host or the resident microbiome. The effect of antiparasitic secondary metabolites on host infections will therefore likely depend on factors like the host genetic background (e.g. regarding enzymes processing secondary compounds), differences in host gene expression of enzymes metabolizing secondary compounds, gut pH [38] and the composition and activity of the microbiome.

Differences in activity against *C. bombi* were driven by changes in glycosylation: for both *Arbutus* and *Tilia* nectar metabolites, aglycones had higher activity than corresponding glycosylated compounds. Similarly, Tasdemir *et al*. [39] found generally higher *in vitro* activity of flavonoid aglycones against several human pathogenic trypanosomatid species than of corresponding glycosides. Glycosylation increases polarity and therefore water solubility of secondary metabolites but decreases their ability to cross cell membranes [40]. Therefore, a possible explanation for the higher antiparasitic activity of the two aglycones studied here is their increased ability to cross *C. bombi* cell membranes relative to their corresponding glycosides. Once in the cytosol of the parasite, the secondary compounds will be able to interfere with cellular processes of the parasite, although exact mechanisms and cellular targets were not studied here. Our findings suggest that antiparasitic effects of glycosylated nectar or pollen metabolites will be underestimated if they can undergo deglycosylation in the host. Tiliaside from linden tree nectar was deglycosylated to the same extent in microbiome-depleted and microbiome-inoculated bumblebees, suggesting that this transformation was dependent on the host, not the microbiome. Bees produce β-glucosidases to catalyse the cleavage of β-glycosidic bonds in the crop and midgut [41], which are likely responsible in our experiment for the deglycosylation. In the case of unedone-8-*O*-β-d-glucoside, deglycosidation in the hindgut was increased in microbiome-inoculated bumblebees, suggesting a role of the microbiome in this case.

More work is needed to understand which members of the microbiome in bees play key roles in the metabolic conversion of secondary metabolites, but experiments by Kešnerová *et al*. [22] on honeybees (*Apis mellifera*) colonized by single strains from the major bacterial phylotypes of the resident microbiome of corbiculate bees suggest a dominant role of lactobacilli (clades ‘Firm-4’ and ‘Firm-5’ *sensu* Martinson *et al*. [42] and bifidobacteria for the deglycosylation of flavonoid glycosides. The genomes of strains from the Firm-5 *Lactobacillus* clade contain a multitude of carbohydrate-processing genes in bumblebees and honeybees (with a higher representation in the latter), including glycosidase hydrolases that could cleave sugars from glycosides [43]. Bifidobacteria from bumblebees and honeybees similarly contain several glycoside hydrolases [44]. Lactobacilli and bifidobacteria are therefore likely candidates for the deglycosidation of the unedone-8-*O*-β-d-glucoside observed in our experiment in the hindgut of bumblebees, but additional experiments are needed to verify this. Both *Lactobacillus* and *Bifidiobacterium* genomes show considerable variation in glycosidase hydrolase contents between strains [43,44]. This suggests the type of bacterial strains a bee is colonized with will influence processing of dietary secondary metabolites, and based on our findings, these strain level variations in the microbiome could, as a consequence, affect parasite persistence or establishment. Perturbation of the microbiome by pollutants, as has been shown for the effect of glyphosate and heavy metals on the honeybee microbiome [45,46], could furthermore indirectly affect parasite success in the bee gut through changes in the metabolism of secondary metabolites by the microbiome.

Conversely, pollinators able to glycosylate secondary metabolites in the gut may have reduced antiparasitic benefits from them (but may benefit from lowered toxicity of the compounds to themselves [47,48]). To our knowledge, our detection of the glycosylation of unedone in the bumblebee midgut (including in microbiome deprived individuals) is the first record of this type of chemical modification of plant secondary metabolites in bees. Further studies are needed to determine how frequently glycosylation of nectar or pollen secondary metabolites occurs in bees and other pollinating insects. Uridine-diphosphate-glycosyl transferases (UGTs) catalyse the glycosylation of xenobiotics in insects and play a role in their detoxification [47,49]. UGTs are present in the genomes of honeybees, bumblebees and solitary bees [18], but only in few (2–12) copies compared with their presence in the genomes of other herbivorous insects such as lepidopterans (over 40 copies; [49]). Mao *et al*. [50] found that ingestion of the pollen secondary metabolite *p*-coumaric acid upregulated the expression of UGTs in honeybees, consistent with their role in secondary metabolite detoxification in this bee species. Health trade-offs for pollinators between the benefits of detoxifying harmful secondary metabolites in the gut by glycosylation and the reduction of antiparasitic activity of glycosylated compounds as suggested in this study are plausible and deserve further attention. Under increased parasite pressure in the host environment, reduced glycosylation rates may be beneficial to limit parasite infections, as benefits from reduced parasite loads may outweigh the costs of damage from toxic secondary metabolites. Whether pollinators can fine-tune glycosylation in this manner in response to parasite risks remains to be investigated.

Lastly, both tree species studied here have major ecological significance as food plants for bumblebees. Strawberry trees (*A. unedo*) are the major autumn food source around the Mediterranean for *B. terrestris* [51], but also serve as late season foraging plants outside of its native range in urban environments, for example for gynes in the UK [29]. *C. bombi* infections have high fitness costs for *B. terrestris* gynes [30], and therefore our observed protection of gynes against *C. bombi* by unedone from *A. unedo* may improve their chances for winter survival and successful nest establishment in the following season. Linden trees (*Tilia* spp.) are major nectar sources in urban environments and temperate deciduous forests [52–54]. Our work emphasizes the potential benefits of trees for pollinators in urban environments, as some species like strawberry and linden trees may provide not only an abundance of food but also health benefits for pollinators. Our findings add to the evidence that plants with antiparasitic (medicinal) activity for pollinators can offer a nature-based solution to maintaining or improving the health of wild pollinators. Positive effects for pollinator health could be achieved both through the conservation or restoration of key medicinal plant species in natural or semi-natural ecosystems [9] and through promoting medicinal plants in managed landscapes, such as agricultural field margins or urban green spaces [11,55]. However, as our understanding of the impacts of nectar and pollen chemistry on wild pollinator health under field conditions remains limited, caution and more research are needed before guidance on landscape-level manipulation of plant species composition for pollinator health can be given [12,56].

## 5. Conclusion

We show that the antiparasitic activity of nectar secondary metabolites can be both increased and decreased during gut passage in a common bumblebee pollinator. This modulation of antiparasitic activity can derive both from the host and from the resident gut microbiome. Effects of secondary metabolites on pollinator parasites therefore cannot necessarily be extrapolated from *in vitro* studies, or studies of a single host–parasite system. Rather, an integrative view of the interaction of the hosts, parasites, secondary metabolites and the resident gut microbiomes needs to be taken for a fuller understanding of the potential benefits of floral reward phytochemicals on pollinator health.

## Data Availability

The data are available in electronic supplementary material, files S1 and S2 [57].

## References

[1] Cameron SA, Lozier JD, Strange JP, Koch JB, Cordes N, Solter LF, Griswold TL. 2011 Patterns of widespread decline in North American bumble bees. Proc. Natl Acad. Sci. USA **108**, 662-667. (10.1073/pnas.1014743108) 21199943 PMC3021065

[2] Schmid-Hempel R et al. 2014 The invasion of southern South America by imported bumblebees and associated parasites. J. Anim. Ecol. **83**, 823-837. (10.1111/1365-2656.12185) 24256429

[3] Fürst MA, McMahon DP, Osborne JL, Paxton RJ, Brown MJF. 2014 Disease associations between honeybees and bumblebees as a threat to wild pollinators. Nature **506**, 364-366. (10.1038/nature12977) 24553241 PMC3985068

[4] Wilfert L, Long G, Leggett HC, Schmid-Hempel P, Butlin R, Martin SJM, Boots M. 2016 Deformed wing virus is a recent global epidemic in honeybees driven by *Varroa* mites. Science **351**, 594-597. (10.1126/science.aac9976) 26912700

[5] Goulson D, Nicholls E, Botías C, Rotheray EL. 2015 Bee declines driven by combined stress from parasites, pesticides, and lack of flowers. Science **347**, 1255957. (10.1126/science.1255957) 25721506

[6] Siviter H, Bailes EJ, Martin CD, Oliver TR, Koricheva J, Leadbeater E, Brown MJF. 2021 Agrochemicals interact synergistically to increase bee mortality. Nature **596**, 389-392. (10.1038/s41586-021-03787-7) 34349259

[7] Richardson LL, Adler LS, Leonard AS, Andicoechea J, Regan KH, Anthony WE, Manson JS, Irwin RE. 2015 Secondary metabolites in floral nectar reduce parasite infections in bumblebees. Proc. R. Soc. B **282**, 20142471. (10.1098/rspb.2014.2471) PMC4345440 25694627

[8] Koch H, Brown MJF, Stevenson PC. 2017 The role of disease in bee foraging ecology. Curr. Opin. Insect Sci. **21**, 60-67. (10.1016/j.cois.2017.05.008) 28822490

[9] Koch H, Woodward J, Langat MK, Brown MJF, Stevenson PC. 2019 Flagellum removal by a nectar metabolite inhibits infectivity of a bumblebee parasite. Curr. Biol. **29**, 3494-3500. (10.1016/j.cub.2019.08.037) 31607528

[10] Bernklau E, Bjostad L, Hogeboom A, Carlisle A, Arathi HS. 2019 Dietary phytochemicals, honey bee longevity and pathogen tolerance. Insects **10**, 14. (10.3390/insects10010014) PMC6359238 30626025

[11] Folly AJ, Koch H, Farrell IW, Stevenson PC, Brown MJF. 2021 Agri-environment scheme nectar chemistry can suppress the social epidemiology of parasites in an important pollinator. Proc. R. Soc. B **288**, 20210363. (10.1098/rspb.2021.0363) PMC8150011 34034519

[12] Sutherland WJ et al. 2020 A horizon scan of emerging global biological conservation issues for 2020. Trends Ecol. Evol. **35**, 81-90. (10.1016/j.tree.2019.10.010) 31813647

[13] Palmer-Young EC et al. 2017 Context-dependent medicinal effects of anabasine and infection-dependent toxicity in bumble bees. PLoS ONE **12**, e0183729. (10.1371/journal.pone.0183729) 28832668 PMC5568382

[14] Thorburn LP, Adler LS, Irwin RE, Palmer-Young EC. 2015 Variable effects of nicotine, anabasine, and their interactions on parasitized bumble bees. F1000Research **4**, 880. (10.12688/f1000research.6870.2)) 26998225 PMC4786900

[15] Palmer-Young EC, Sadd BM, Irwin RE, Adler LS. 2017 Synergistic effects of floral phytochemicals against a bumble bee parasite. Ecol. Evol. **7**, 1836-1849. (10.1002/ece3.2794) 28331591 PMC5355193

[16] Palmer-Young EC, Sadd BM, Stevenson PC, Irwin RE, Adler LS. 2016 Bumble bee parasite strains vary in resistance to phytochemicals. Scient. Rep. **6**, 37087. (10.1038/srep37087) PMC5121629 27883009

[17] Vidkjær NH, Fomsgaard IS, Kryger P. 2021 LC–MS/MS quantification reveals ample gut uptake and metabolization of dietary phytochemicals in honey bees (*Apis mellifera*). J. Agric. Food Chem. **69**, 627-637. (10.1021/acs.jafc.0c03584) 33416324 PMC7884015

[18] Berenbaum MR, Johnson RM. 2015 Xenobiotic detoxification pathways in honey bees. Curr. Opin. Insect Sci. **10**, 51-58. (10.1016/j.cois.2015.03.005) 29588014

[19] du Rand EE, Smit S, Beukes M, Apostolides Z, Pirk CWW, Nicolson SW. 2015 Detoxification mechanisms of honey bees (*Apis mellifera*) resulting in tolerance of dietary nicotine. Scient. Rep. **5**, 11779. (10.1038/srep11779) PMC4488760 26134631

[20] Sadd BM et al. 2015 The genomes of two key bumblebee species with primitive eusocial organization. Genome Biol. **16**, 76. (10.1186/s13059-015-0623-3) 25908251 PMC4414376

[21] Kwong WK, Medina LA, Koch H, Sing KW, Soh EJY, Ascher JS, Jaffé R, Moran NA. 2017 Dynamic microbiome evolution in social bees. Sci. Adv. **3**, e1600513. (10.1126/sciadv.1600513) 28435856 PMC5371421

[22] Kešnerová L, Mars RA, Ellegaard KM, Troilo M, Sauer U, Engel P. 2017 Disentangling metabolic functions of bacteria in the honey bee gut. PLoS Biol. **15**, e2003467. (10.1371/journal.pbio.2003467) 29232373 PMC5726620

[23] Koch H, Schmid-Hempel P. 2011 Socially transmitted gut microbiota protect bumble bees against an intestinal parasite. Proc. Natl Acad. Sci. USA **108**, 19 288-19 292. (10.1073/pnas.1110474108) PMC3228419 22084077

[24] Koch H, Schmid-Hempel P. 2012 Gut microbiota instead of host genotype drive the specificity in the interaction of a natural host-parasite system. Ecol. Lett. **15**, 1095-1103. (10.1111/j.1461-0248.2012.01831.x) 22765311

[25] Mockler BK, Kwong WK, Moran NA, Koch H. 2018 Microbiome structure influences infection by the parasite *Crithidia bombi* in bumble bees. Appl. Environ. Microbiol. **84**, e02335-17. (10.1128/AEM.02335-17) 29374030 PMC5861814

[26] Naef R, Jaquier A, Velluz A, Bachofen B. 2004 From the linden flower to linden honey – volatile constituents of linden nectar, the extract of bee-stomach and ripe honey. Chem. Biodivers. **1**, 1870-1879. (10.1002/cbdv.200490143) 17191825

[27] Frérot E, Velluz A, Decorzant E, Naef R. 2006 From linden flower to linden honey. Part 2: glycosidic precursors of cyclohexa-1,3-diene-1-carboxylic acids. Chem. Biodivers. **3**, 94-100. (10.1002/cbdv.200690012) 17193221

[28] Tuberoso CI, Bifulco E, Caboni P, Cottiglia F, Cabras P, Floris I. 2010 Floral markers of strawberry tree (*Arbutus unedo* L.) honey. J. Agric. Food Chem. **58**, 384-389. (10.1021/jf9024147) 19919097

[29] Stelzer RJ, Chittka L, Carlton M, Ings TC. 2010 Winter active bumblebees (*Bombus terrestris*) achieve high foraging rates in urban Britain. PLoS ONE **5**, e9559. (10.1371/journal.pone.0009559) 20221445 PMC2832779

[30] Brown MJF, Schmid-Hempel R, Schmid-Hempel P. 2003 Strong context-dependent virulence in a host–parasite system: reconciling genetic evidence with theory. J. Anim. Ecol. **72**, 994-1002. (10.1046/j.1365-2656.2003.00770.x)

[31] R Core Team. 2021 R: a language and environment for statistical computing. R Foundation for Statistical Computing. Vienna, Austria. See https://www.R-project.org/.

[32] Brown MJF, Loosli R, Schmid-Hempel P. 2000 Condition-dependent expression of virulence in a trypanosome infecting bumblebees. Oikos **91**, 421-427. (10.1034/j.1600-0706.2000.910302.x)

[33] Pinheiro J, Bates D, DebRoy S, Sarkar D, R Core Team. 2021 *nlme: Linear and nonlinear mixed effects models*. *R package version 3.1-153*. See https://CRAN.R-project.org/package=nlme.

[34] Salathe R, Tognazzo M, Schmid-Hempel R, Schmid-Hempel P. 2012 Probing mixed-genotype infections I: extraction and cloning of infections from hosts of the trypanosomatid *Crithidia bombi*. PLoS ONE **7**, e49046. (10.1371/journal.pone.0049046) 23155449 PMC3498296

[35] Ritz C, Baty F, Streibig JC, Gerhard D. 2015 Dose-response analysis using R. PLoS ONE **10**, e0146021. (10.1371/journal.pone.0146021) 26717316 PMC4696819

[36] Tian L, Hines HM. 2018 Morphological characterization and staging of bumble bee pupae. PeerJ **6**, e6089. (10.7717/peerj.6089) 30588402 PMC6302898

[37] Langat LC, Langat MK, Wetschnig W, Knirsch W, Mulholland DA. 2021 Antiproliferative bufadienolides from the bulbs of *Drimia altissima*. J. Nat. Prod. **84**, 608-615. (10.1021/acs.jnatprod.0c01079) 33478223

[38] Poreddy S, Mitra S, Schöttner M, Chandran J, Schneider B, Baldwin IT, Kumar P, Pandit SS. 2015 Detoxification of hostplant's chemical defence rather than its anti-predator co-option drives β-glucosidase-mediated lepidopteran counteradaptation. Nat. Commun. **6**, 8525. (10.1038/ncomms9525) 26443324 PMC4633822

[39] Tasdemir D, Kaiser M, Brun R, Yardley V, Schmidt TJ, Tosun F, Rüedi P. 2006 Antitrypanosomal and antileishmanial activities of flavonoids and their analogues: *in vitro*, *in vivo*, structure-activity relationship, and quantitative structure-activity relationship studies. Antimicrob. Agents Chemother. **50**, 1352-1364. (10.1128/AAC.50.4.1352-1364.2006) 16569852 PMC1426963

[40] Vasudevan UM, Lee EY. 2020 Flavonoids, terpenoids, and polyketide antibiotics: role of glycosylation and biocatalytic tactics in engineering glycosylation. Biotechnol. Adv. **41**, 107550. (10.1016/j.biotechadv.2020.107550) 32360984

[41] Pontoh J, Low NH. 2002 Purification and characterization of β-glucosidase from honey bees (*Apis mellifera*). Insect Biochem. Mol. Biol. **32**, 679-690. (10.1016/S0965-1748(01)00147-3) 12020842

[42] Martinson VG, Danforth BN, Minckley RL, Rueppell O, Tingek S, Moran NA. 2011 A simple and distinctive microbiota associated with honey bees and bumble bees. Mol. Ecol. **20**, 619-628. (10.1111/j.1365-294X.2010.04959.x) 21175905

[43] Ellegaard KM et al. 2019 Genomic changes underlying host specialization in the bee gut symbiont *Lactobacillus* Firm5. Mol. Ecol. **28**, 2224-2237. (10.1111/mec.15075) 30864192

[44] Zheng H, Perreau J, Powell JE, Han B, Zhang Z, Kwong WK, Tringe SG, Moran NA. 2019 Division of labor in honey bee gut microbiota for plant polysaccharide digestion. Proc. Natl Acad. Sci. USA **116**, 25 909-25 916. (10.1073/pnas.1916224116) PMC6926048 31776248

[45] Motta EV, Raymann K, Moran NA. 2018 Glyphosate perturbs the gut microbiota of honey bees. Proc. Natl Acad. Sci. USA **115**, 10 305-10 310. (10.1073/pnas.1803880115) PMC6187125 30249635

[46] Rothman JA, Leger L, Kirkwood JS, McFrederick QS. 2019 Cadmium and selenate exposure affects the honey bee microbiome and metabolome, and bee-associated bacteria show potential for bioaccumulation. Appl. Environ. Microbiol. **85**, e01411-19. (10.1128/AEM.01411-19) 31471302 PMC6803295

[47] Despres L, David JP, Gallet C. 2007 The evolutionary ecology of insect resistance to plant chemicals. Trends Ecol. Evol. **22**, 298-307. (10.1016/j.tree.2007.02.010) 17324485

[48] Heckel DG. 2018 Insect detoxification and sequestration strategies. Annu. Plant Rev. **47**, 77-114. (10.1002/9781119312994.apr0507)

[49] Ahn SJ, Vogel H, Heckel DG. 2012 Comparative analysis of the UDP-glycosyltransferase multigene family in insects. Insect Biochem. Mol. Biol. **42**, 133-147. (10.1016/j.ibmb.2011.11.006) 22155036

[50] Mao W, Schuler MA, Berenbaum MR. 2013 Honey constituents up-regulate detoxification and immunity genes in the western honey bee *Apis mellifera*. Proc. Natl Acad. Sci. USA **110**, 8842-8846. (10.1073/pnas.1303884110) 23630255 PMC3670375

[51] Rasmont P et al. 2005 Analysis of pollen and nectar of *Arbutus unedo* as a food source for *Bombus terrestris* (Hymenoptera: Apidae). J. Econ. Entomol. **98**, 656-663. (10.1603/0022-0493-98.3.656) 16022289

[52] Somme L, Moquet L, Quinet M, Vanderplanck M, Michez D, Lognay G, Jacquemart AL. 2016 Food in a row: urban trees offer valuable floral resources to pollinating insects. Urban Ecosyst. **19**, 1149-1161. (10.1007/s11252-016-0555-z)

[53] Koch H, Stevenson PC. 2017 Do linden trees kill bees? Reviewing the causes of bee deaths on silver linden (*Tilia tomentosa*). Biol. Lett. **13**, 20170484. (10.1098/rsbl.2017.0484) 28954857 PMC5627179

[54] Stevenson PC et al. 2020 The state of the world's urban ecosystems: what can we learn from trees, fungi, and bees? Plants People Planet **2**, 482-498. (10.1002/ppp3.10143)

[55] Giacomini JJ, Leslie J, Tarpy DR, Palmer-Young EC, Irwin RE, Adler LS. 2018 Medicinal value of sunflower pollen against bee pathogens. Scient. Rep. **8**, 14394. (10.1038/s41598-018-32681-y) PMC6158195 30258066

[56] Brown MJF. 2022 Complex networks of parasites and pollinators: moving towards a healthy balance. Phil. Trans. R. Soc. B **377**, 20210161. (10.1098/rstb.2021.0161) PMC9058525 35491603

[57] Koch H, Welcome V, Kendal-Smith A, Thursfield L, Farrell IW, Langat MK, Brown MJF, Stevenson PC. 2022 Host and gut microbiome modulate the antiparasitic activity of nectar metabolites in a bumblebee pollinator. Figshare. (10.6084/m9.figshare.c.5912833) PMC9058528 35491601

